# Editorial: Atypical and malignant meningioma: Advances in pathophysiology, imaging and treatment

**DOI:** 10.3389/fneur.2022.970394

**Published:** 2022-08-16

**Authors:** Maleeha Ahmad, Antonio Meola, Erqi Pollom

**Affiliations:** ^1^Department of Neurosurgery, Stanford University School of Medicine, Palo Alto, CA, United States; ^2^Department of Radiation Oncology, Stanford University School of Medicine, Palo Alto, CA, United States

**Keywords:** meningioma, atypical meningioma, malignant meningioma, radiomics, brain tumor

Atypical meningiomas are a heterogeneous group of tumors, different to the benign WHO grade 1 meningiomas, and with sub-group varying levels of aggression and recurrence, making prognostication, and thereby management, often complicated for the clinician and the patient. In this edition of Frontiers, the Editors have selected key publications which may facilitate decision making for surveillance, treatment options, and even prognostication modeling using AI.

As we march through the twenty-first century with significant technological progress, specifically with reference to applications of artificial intelligence, the overlap with our partners in Molecular Biology, Radiology, Medical Physics, and Computational Biology becomes even more remarkable. An ode to this is the study by Xiao et al., in which the authors utilized radiological features to pre-operatively predict brain invasion. As Neurosurgeons and clinicians, we routinely review all the radiological features the authors have used as objective measures in their radiological cohort—peritumoral edema as seen on FLAIR and post-contrast architecture of the tumor on MR. The use of an open source github Python package, in addition to the pathological confirmation of brain invasion and additional blood indices, leads to the extrapolation of this multi-faceted study of how a conglomeration of clinical factors may in the future lead to AI-hybrid decision-making for the optimal care of our patients.

Millesi et al.'s study is of keen interest here, showing the potential strong prognostication role of DNA methylation in the often unpredictable course of WHO grade 2 meningiomas. I bring the reader's attention to the powerful heatmap in Figure 4 showing the correlation of DNA methylation patterns in response to clinical patterns and outcomes. While this is a small cohort of patients, the methylation was associated with a poor prognostic course and surprisingly, showed no association with meningioma location size, Simpson grade, or predicted meningioma methylation class.

Continuing on the molecular biology theme, the case report by Hong et al. follows the therapeutic journey of a patient with recurrent and refractory skull base meningioma, and how the molecular markers, specifically of *GNAS* mutation, led to the multi-disciplinary team widening their treatment options to include tyrosine kinase inhibitor sunitinib.

The fourth publication in this Frontiers series is the impressive work undertaken by Gao et al. looking at the relationship of a vast array of hematological markers related to the progression and potential prognostication of WHO grade 2 meningiomas. For researchers interested in establishing or refining robust clinical prognostication models, I refer you to the authors' detailed work in Figures 2A,B.

We hope our colleagues and readers find this Research Topic to be of as much educational interest and to awaken as much academic fervor as it did for myself and my colleagues during the review of these manuscripts.

## Author contributions

MA, AM, and EP contributed to conception and design of this journal, reviewed the submitted manuscripts, and designated the Reviewers. All authors contributed to manuscript revision, read, and approved the submitted version.

## Conflict of interest

The authors declare that the research was conducted in the absence of any commercial or financial relationships that could be construed as a potential conflict of interest.

## Publisher's note

All claims expressed in this article are solely those of the authors and do not necessarily represent those of their affiliated organizations, or those of the publisher, the editors and the reviewers. Any product that may be evaluated in this article, or claim that may be made by its manufacturer, is not guaranteed or endorsed by the publisher.

